# A Visual Task Management Application for Acute Ischemic Stroke Care

**DOI:** 10.3389/fneur.2019.01118

**Published:** 2019-10-30

**Authors:** Shoji Matsumoto, Hiroshi Koyama, Ichiro Nakahara, Akira Ishii, Taketo Hatano, Tsuyoshi Ohta, Koji Tanaka, Mitsushige Ando, Hideo Chihara, Wataru Takita, Keisuke Tokunaga, Takuro Hashikawa, Yusuke Funakoshi, Takahiko Kamata, Eiji Higashi, Sadayoshi Watanabe, Daisuke Kondo, Atsushi Tsujimoto, Konosuke Furuta, Takuma Ishihara, Tetsuya Hashimoto, Junpei Koge, Kazutaka Sonoda, Takako Torii, Hideaki Nakagaki, Ryo Yamasaki, Izumi Nagata, Jun-ichi Kira

**Affiliations:** ^1^Department of Comprehensive Strokology, Fujita Health University School of Medicine, Toyoake, Japan; ^2^Graduate School of Industrial Technology, Advanced Institute of Industrial Technology, Shinagawa, Japan; ^3^Department of Neurosurgery, Kyoto University Hospital, Kyoto, Japan; ^4^Department of Neurosurgery, Kokura Memorial Hospital, Kitakyushu, Japan; ^5^Department of Neurosurgery, Kochi Health Sciences Center, Kochi, Japan; ^6^Department of Neurology, Graduate School of Medical Sciences, Neurological Institute, Kyushu University, Fukuoka, Japan; ^7^Department of Neurosurgery, Shiga General Hospital, Moriyama, Japan; ^8^Department of Neurology, National Hospital Organization Nagoya Medical Center, Nagoya, Japan; ^9^Department of Cerebrovascular Medicine and Neurology, National Hospital Organization Kyushu Medical Center, Fukuoka, Japan; ^10^Department of Neurosurgery, St. Mary's Hospital, Kurume, Japan; ^11^Department of Neurosurgery, Kobe City Medical Center General Hospital, Kobe, Japan; ^12^Department of Cerebrovascular Medicine, Saga Medical Centre Koseikan, Saga, Japan; ^13^Department of Neurology, Japan Community Health Care Organization Kyushu Hospital, Kitakyushu, Japan; ^14^Department of Neurology, Kokura Memorial Hospital, Kitakyushu, Japan; ^15^Innovative and Clinical Research Promotion Center, Gifu University Hospital, Gifu, Japan; ^16^Department of Neurology, University of California, Los Angeles, Los Angeles, CA, United States; ^17^Department of Cerebrovascular Medicine, National Cerebral and Cardiovascular Center, Osaka, Japan; ^18^Department of Neurology, Saiseikai Fukuoka General Hospital, Fukuoka, Japan; ^19^Department of Neurology and Neuroscience, Nagoya City University Graduate School of Medical Sciences, Nagoya, Japan; ^20^Department of Neurology, Fukuoka City Hospital, Fukuoka, Japan

**Keywords:** acute ischemic stroke, endovascular therapy, intravenous thrombolysis, processing times, visual task management

## Abstract

**Background:** To maximize the effect of intravenous (IV) thrombolysis and/or endovascular therapy (EVT) for acute ischemic stroke (AIS), stroke centers need to establish a parallel workflow on the basis of a code stroke (CS) protocol. At Kokura Memorial Hospital (KMH), we implemented a CS system in January 2014; however, the process of information sharing within the team has occasionally been burdensome.

**Objective:** To solve this problem using information communication technology (ICT), we developed a novel application for smart devices, named “Task Calc. Stroke” (TCS), and aimed to investigate the impact of TCS on AIS care.

**Methods:** TCS can visualize the real-time progress of crucial tasks for AIS on a dashboard by changing color indicators. From August 2015 to March 2017, we installed TCS at KMH and recommended its use during normal business hours (NBH). We compared the door-to-computed tomography time, the door-to-complete blood count (door-to-CBC) time, the door-to-needle for IV thrombolysis time, and the door-to-puncture for EVT time among three treatment groups, one using TCS (“TCS-based CS”), one not using TCS (“phone-based CS”), and one not based on CS (“non-CS”). A questionnaire survey regarding communication problems was conducted among the CS teams at 3 months after the implementation of TCS.

**Results:** During the study period, 74 patients with AIS were transported to KMH within 4.5 h from onset during NBH, and 53 were treated using a CS approach (phone-based CS: 26, TSC-based CS: 27). The door-to-CBC time was significantly reduced in the TCS-based CS group compared to the phone-based CS group, from 31 to 19 min (*p* = 0.043). Other processing times were also reduced, albeit not significantly. The rate of IV thrombosis was higher in the TCS-based CS group (78% vs. 46%, *p* = 0.037). The questionnaire was correctly filled in by 34/38 (89%) respondents, and 82% of the respondents felt a reduction in communication burden by using the TCS application.

**Conclusions:** TCS is a novel approach that uses ICT to support information sharing in a parallel CS workflow in AIS care. It shortens the processing times of critical tasks and lessens the communication burden among team members.

## Introduction

Early administration of intravenous tissue plasminogen activator (IV-tPA) and/or endovascular therapy (EVT) is associated with improved outcomes in acute ischemic stroke (AIS) (1, 2). Guidelines recommend a time interval from patient arrival to start of IV-tPA administration, known as the door-to-needle (DTN) time, of ≤ 30–60 min (3, 4). The “Target: Stroke” project, involving leading stroke centers, succeeded in reducing DTN times by the implementation of a code stroke (CS) algorithm (5, 6).

In a stroke center, many tasks need to be performed across multiple departments in order to quickly decide on the therapeutic indication for IV-tPA or EVT (3, 4). The key concept in fast treatment is the paradigm of parallel (rather than serial) diagnostic evaluation, assessment, and treatment (7–9), which requires a high commitment among all stroke team members in the organizational structure (10).

At Kokura Memorial Hospital (KMH), we have attempted to shorten DTN times since January 2014, by designating a professional team in the organizational structure and implementing a 24/7 CS system consisting of a pre-arrival notification and activation of the entire stroke team by phone, rapid laboratory testing, an uninterrupted supply of the IV-tPA tool kit, and continuous availability of computed tomography (CT) and magnetic resonance imaging (MRI). In 2014, the annual median DTN time was reduced from around 90 min in the previous year to about 40 min.

However, new problems have appeared after the implementation of CS. When CS is initiated, various team members from several divisions of the hospital have to be contacted by phone. Communication delays occasionally occur when the emergency department (ED) is crowded and staff cannot call immediately, or when phone lines are busy. Moreover, during normal business hours (NBH), defined as the period of time in the hospital from 08:30 until 17:00 on the same day from Monday through Friday, various in-hospital procedures, such as laboratory testing, CT, MRI, and angiography, are booked out with pre-scheduled appointments. While the algorithm was running, team members had to coordinate by making many phone calls among themselves, which was sometimes perceived as a significant burden on the team. However, because the accurate arrival time of a patient at a test site is often unknown, tests are often delayed, resulting in extended DTN times.

To solve these problems regarding the sharing of information by an organized CS team using information and communication technology (ICT), we developed “Task Calc. Stroke” (TCS), a visual management application for smart devices (11), and installed it at KMH in August 2015. We then evaluated the impact of TCS on critical task processing times in AIS care and the rate of treated IV-tPA and EVT during the trial period from August 2015 until March 2017. Our study hypothesis was that using TCS will shorten key processing times in AIS care and reduce the burden of information sharing within the team.

## Materials and Methods

**Figure 1:**
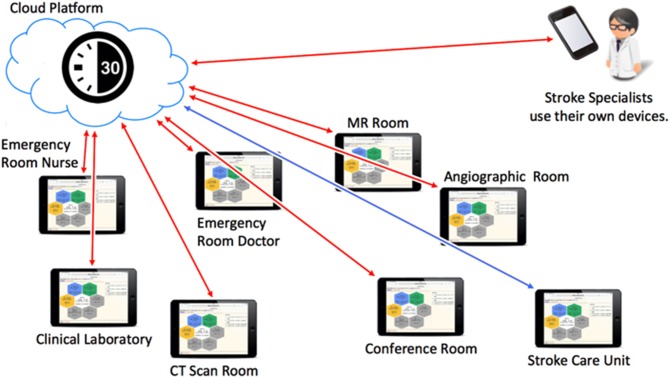
Concept image of “Task Calc. Stroke” (TCS).

TCS is an in-house application for smart devices or personal computers that is implemented via the internet. On August 1, 2015, using commercially available smart devices (iPad or iPad mini; Apple, Cupertino, CA, USA), TCS was installed in the emergency room (ER), CT scan room, MRI room, angiographic room, clinical laboratory room, stroke care unit, and conference room at KMH. These devices were used in a static manner and required personnel to remain at a specific place; hence, we used TCS only during NBH. The stroke specialists also accessed TCS from their own devices (see Figure 1).

From August 2015 to March 2017, we recommended the use of TCS to ED doctors when CS was initiated for patients with suspected AIS who arrived at KMH within 4.5 h from onset during NBH.

**Figure 2:**
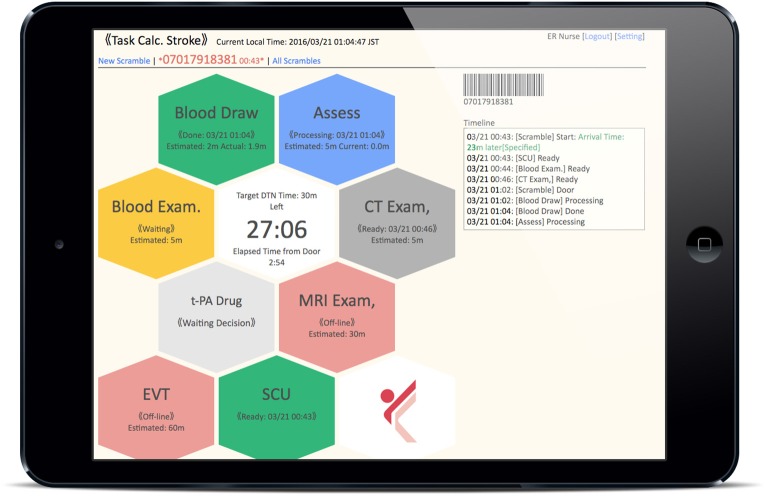
Dashboard of Task Calc. Stroke (TCS). All of the “tasks to do” for acute ischemic stroke therapy assigned to medical staff in each department are displayed as colored hexagons on the TCS dashboard.

**Figure 3:**
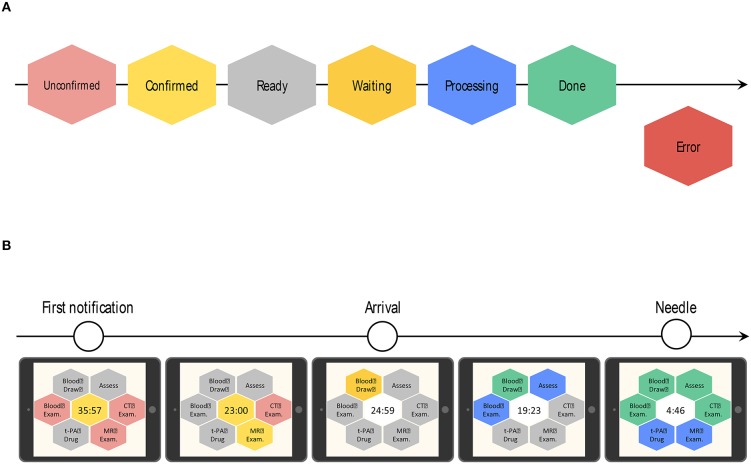
Color-based indication of task progress and the transition of the countdown timer on the dashboard of Task Calc. Stroke (TCS). **(A)** Hexagon colors indicate the progress status of each task. After clicking the “tasks to do” icon, the real-time progress of each task is indicated by a change in color, as follows: unconfirmed patient arrival notification (light red), confirmed patient arrival notification (yellow), completed patient preparation (gray), processing patient task (blue), completed patient task (green), and error (red). **(B)** The countdown timer is located at the center of the dashboard. TCS automatically calculates the time to patient arrival. Before patient arrival, it displays the remaining time based on the estimated patient arrival time on a yellow background. After the patient arrives, the timer shows the remaining time according to the preset door-to-needle (DTN) time on a white background.

**Figure 4:**
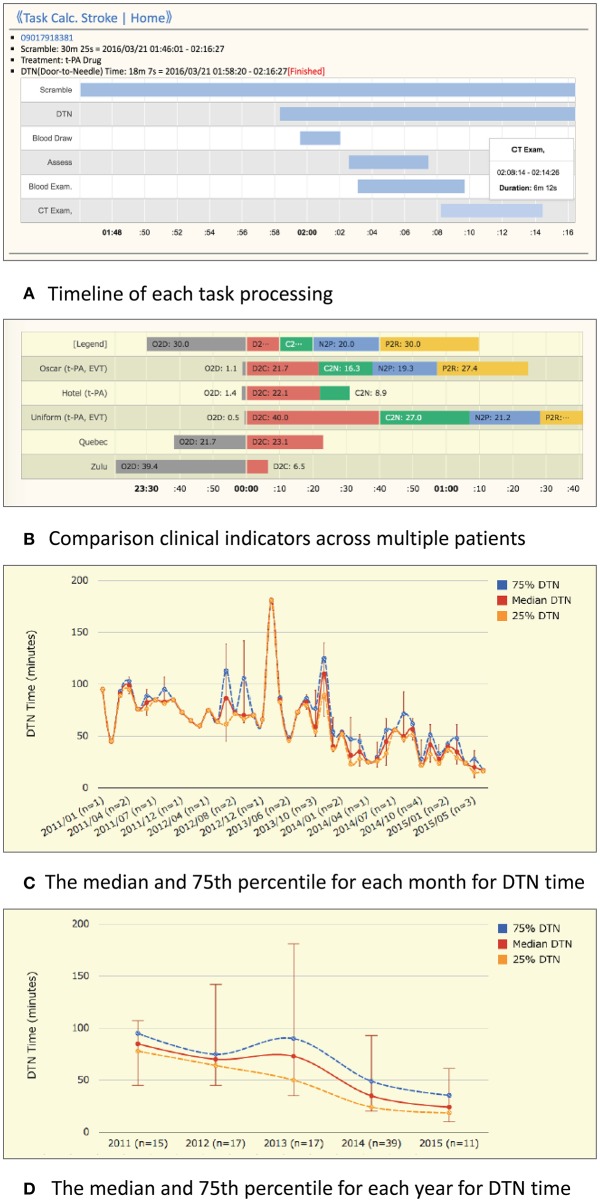
Example charts for the performance history analyzer function of Task Calc. Stroke (TCS). **(A)** An example chart of the timeline overview of a patient who received treatment for intravenous thrombolysis. An overlap of tasks and the waiting time for each task can be identified easily. **(B)** An example chart consists of onset-to-door (O2D), door-to-CT (D2C), CT-to-needle (D2N), needle-to-puncture (N2P), CT-to-puncture (CT2P), and puncture-to-reperfusion (P2R) times. The differences in time metrics among patients can be visually compared. **(C)** Monthly average view: an example graph of the maximum, 75%, median, 25%, and minimum DTN times in 1 month. **(D)** Yearly average view: an example graph of the maximum, 75%, median, 25%, and minimum DTN times in 1 year.

TCS enables parallel CS workflow by visualizing the progress of the multiple tasks involved in AIS care and sharing them among the medical staff. When TCS is not used, for realizing parallel workflow, medical staff need to make various phone calls or use instant messaging applications among the team. TCS visually supports four main functions as described in the following:

- *Notifications:* When the ED doctor receives an emergency pre-arrival notification, he/she can quickly notify all stroke team members of the arrival of a suspected stroke patient and of the estimated arrival time, with a single click on the device. Then, the TCS located in each department makes a loud sound and indicates that a patient is being transported.
- *Color recognition:* In line with the workflow of the CS that started in 2014 at KMH, critical tasks assigned to medical staff in each department (nurses, doctors, radiology technicians, laboratory technicians) are displayed as colored hexagons in order of priority on the TCS dashboard (Figure 2). After clicking on the “tasks to do” icon, the current status and progress of each task is indicated by a change in color, as follows: unconfirmed patient arrival notification (light red), confirmed patient arrival notification (yellow), completed patient preparation (gray), processing patient task (blue), completed patient task (green), and error (red) (Figure 3A). For example, when a laboratory technician initiates a blood examination and presses the status button “being processed,” a blue hexagon is displayed. In the same manner, when blood examination is finished and the status button “done” is pressed, a green hexagon is displayed. At every moment, the dashboard displays the progress information for a particular patient, and all stroke team members can perform their tasks while seeing the real-time progress of other departments involved in AIS care. If a stroke specialist knows that a blood test has begun, he or she can predict when the test results will be available. Furthermore, if the preparation status of CT, MR, or angiography is known, team members can immediately judge whether tPA or EVT can be administered. Clinical laboratory, CT, MRI, and angiographic technicians can estimate when a blood sample or a patient will arrive. As a result, pre-scheduled appointments can be adjusted to minimize waiting times for the examinations. Oversights or delays in preparations can be quickly identified and corrected. The number and types of task hexagons can be customized depending on the specific protocol for AIS care.
- *Synchronized timers:* TCS has synchronized timers that are arranged in the center of the dashboard. From pre-arrival notification to patient arrival, TCS displays the remaining time relative to the estimated arrival time on a yellow background. After arrival, the remaining time according to the preset DTN time is displayed on a white background (Figure 3B). Each hexagon also has an individual timer that analyzes the individual time course of each task.
- *Performance history analysis*: TCS automatically records processing time data and displays timelines and charts for every case and for some clinical indicators across multiple patients. Figure 4A shows an example of the timeline overview for a patient who received treatment for intravenous thrombolysis. Overlaps and waiting times for each task are indicated. Crucial indicators such as onset-to-door (O2D), door-to-CT (D2C), CT-to-needle (CT2N), needle-to-puncture (N2P), CT-to-puncture (CT2P), and puncture-to-reperfusion (P2R) times can be visualized and compared among patients (Figure 4B). TCS also allows the visualization of changes in DTN times over months (Figure 4C) and years (Figure 4D).

### Evaluation

We retrospectively reviewed the data from consecutive patients with AIS transported within 4.5 h of symptom onset and divided them into three groups: a group where the CS system was not activated (“non-CS”), a group where CS was used without TCS (“phone-based CS”), and a group where the CS system and the TCS application were used (“TCS-based CS”). To investigate the effect of TCS on AIS care, we compared D2C times, door-to-complete blood count (CBC; including platelet count) times, DTN times for IV-tPA, and door-to-puncture times for EVT between the TCS-based CS and phone-based CS groups of patients transported during NBH. We also compared IV-tPA and EVT rates between the TCS-based CS and phone-based CS groups of patients transported during NBH.

To investigate the effect of CS itself, we compared D2C times, door-to-CBC times, DTN times, and D2P times between the non-CS and phone-based CS or TCS-based CS groups.

We conducted a questionnaire survey regarding communication problems in the workplace among the team at 3 months after the implementation of TCS.

The ethics committee of KMH approved this study. Opt-out consent was obtained instead of written informed consent. We provided patients with information explaining the proposed research plan (the purpose, required individual data, and duration of the study) via the website of KMH and gave them the opportunity to opt out at any point during the study. All procedures were in accordance with the Helsinki Declaration of 2000 and the Declaration of Istanbul of 2008.

### Sample Size

The study sample size was determined according to feasibility and to avoid overfitting. Since we planned to use four covariates in the primary analysis, at least 60 patients were required (12). A final sample size of 74 patients allowed us to assess the study outcomes, at 82% power (empirical power) at a two-sided significance level of 5% (with D2C times of 15.81 vs. 36.02 min for the TCS-based CS and non-TCS based CS (phone-based CS and non-CS) and a pooled standard deviation of 28.94 min.

### Statistical Analysis

Data are expressed as numbers and percentages, or as mean values ± standard deviations. Statistical analyses were performed using the statistical software SPSS version 11.0 (SPSS Inc., Chicago, IL, USA) and R version 3.5.1 (http://www.r-project.org). For the primary analysis, multiple linear regression models were used to assess independent associations between the treatment groups (TCS-based CS, phone-based CS, non-CS) during NBH and key task processing times (D2C time, door-to-CBC time, DTN time for IV-tPA, door-to-puncture (D2P) time for EVT) with adjustments for large vessel occlusion (LVO), oral anticoagulants, and the initial National Institutes of Health Stroke Scale (NIHSS) score as a confounder. For the secondary analysis, effects of the treatment (phone-based CS, non-CS) outside of NBH on key task processing times were also assessed using a multiple linear regression model adjusted for the abovementioned confounders. To assess the effects of TCS on IV-tPA and EVT for CS, a multivariable logistic regression model was used adjusted for LVO, oral anticoagulants, and the initial NIHSS score as a confounder. To assess the dispersion of key time metrics, the variance estimators and their confidence intervals (CIs) were used. Distributions of key time metrics were distorted to the left, and outcomes were thus natural log-transformed to ensure normality in regression residuals. An F-test was performed to compare the variance between the two groups. A *p*-value <0.05 was considered statistically significant for all analyses.

## Results

A total of 183 patients with AIS arrived at KMH within 4.5 h of symptom onset during the study period. The patients' clinical characteristics are shown in Table 1. A total of 74/183 (40%) patients were transported during NBH, and 53/74 (72%) patients were treated based on the CS system (27 patients were treated with TCS, and the remaining 26 patients were treated without TCS). A total of 109/183 (60%) patients were transported outside of NBH, and 55/109 (50%) patients were treated with CS without TCS. Our trial installation of TCS showed the successful sharing of information.

**Table 1:** Patient characteristics.

	**NBH (*****N*** **=** **74)**			**Outside NBH (*****N*** **=** **109)**	
	**TCS-based CS**	**Phone-based CS**	**Non-CS**	**Phone-based CS**	**Non-CS**
	**(*N* = 27)**	**(*N* = 26)**	**(*N* = 21)**	**(*N* = 55)**	**(*N* = 54)**
Age, *y*, mean (SD)	75.0 (69.0, 85.0)	77.0 (64.3, 82.8)	80.0 (72.0, 87.0)	76.0 (66.5, 84.0)	75.0 (66.3, 84.0)
Sex, male, *n* (%)	17 (63)	14 (53)	12 (57)	33 (60)	35 (65)
Atrial fibrillation, *n* (%)	10 (37)	10 (39)	8 (38)	28 (51)	28 (52)
Dyslipidemia, *n* (%)	7 (26)	11 (42)	7 (33)	19 (35)	23 (43)
Hypertension, *n* (%)	16 (59)	14 (54)	14 (67)	33 (60)	35 (65)
Diabetes mellitus, *n* (%)	5 (19)	7 (27)	4 (19)	12 (22)	13 (24)
Onset-to-door time, min (SD)	39.0 (32.5, 74.5)	47.0 (31.0, 68.3)	71.0 (55.0, 139.0)	54.0 (35.0, 79.5)	72.0 (43.0, 132.0)
NIHSS at admission, mean (SD)	10.0 (3.5, 15.0)	6.0 (3.3, 15.8)	2.0 (0.0, 4.0)	6.0 (1.5, 20.5)	5.0 (2.0, 12.8)
Large vessel occlusion, *n* (%)	11 (41)	8 (31)	3 (14)	26 (47)	15 (28)
Oral anticoagulants *n* (%)	3 (11)	5 (19)	5 (24)	14 (26)	10 (19)

*n, number; y, years; SD, standard deviation; TCS, Task Calc. Stroke; CS, code stroke; NIHSS, National Institutes of Health Stroke Scale, NBH, normal business hours*.

### Results of the Effects of TCS on AIS Care

**Figure 5:**
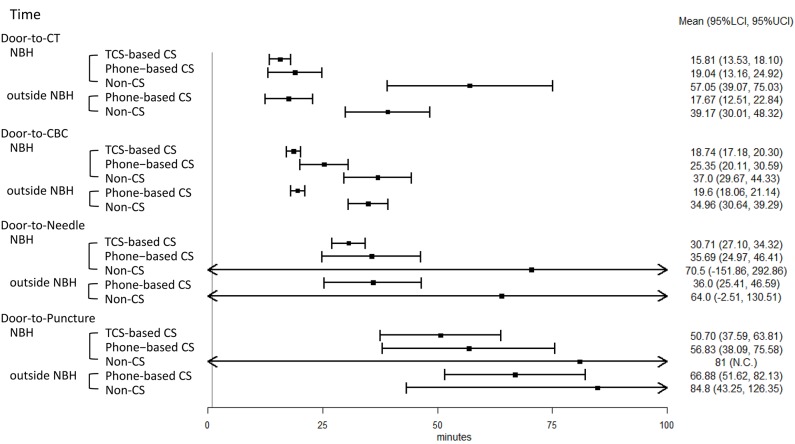
Forest plot of key task processing times in acute ischemic stroke (AIS) care. NBH, normal business hours; TCS, Task Calc. Stroke; CS, code stroke; CT, computed tomography; CBC, complete blood count; IV-tPA, intravenous tissue plasminogen activator; EVT, endovascular therapy; LCI, lower 95% confidence interval; UCI, upper 95% confidence interval; NC, not calculated because there is only one sample.

The key task processing times are shown in Figure 5. The door-to-CBC time was significantly reduced in the TCS-based CS group compared to the phone-based CS group, after adjustments for LVO, prior anticoagulation therapy, and the initial NIHSS score (Table 2). With TCS-based CS, the door-to-CBC time decreased from 31 min (95% CI: 13–25) to 19 min [14–18] (β = 0.81 [0.66–0.99], *p* = 0.043). Other processing times were also reduced but not significantly. The rate of IV-tPA was higher in the TSC-based CS than in the phone-based CS group (78% vs. 46%) (Table 3). A multivariable logistic regression model showed that TCS-based CS was independently associated with IV-tPA (odds ratio 3.98, 95% CI: 1.08–14.59, *p* = 0.037) (Table 4). The rate of EVT was almost twofold in the TCS-based CS group compared to the phone-based CS group; however, a multivariable logistic regression model showed that TCS-based CS was not associated with EVT (odds ratio 7.85, 95% CI: 0.4–153.48, *p* = 0.174) (Tables 3, 4).

**Table 2:** Comparisons of task processing times between the three group (TCS-based CS vs. phone-based CS vs. non-CS) during NBH and two groups (phone-based CS vs. non-CS) outside of NBH.

**Key task processing times**		**β**	**95% CI**	***p*-value**
**Door-to-CT**				
NBH	TCS-based CS vs. phone-based CS	0.915	[0.711, 1.178]	0.487
	TCS-based CS vs. non-CS	0.33	[0.25, 0.437]	<0.001
	Phone-based CS vs. non-CS	0.361	[0.275, 0.473]	<0.001
Outside NBH	Phone-based CS vs. non-CS	0.519	[0.415, 0.649]	<0.001
**Door-to-CBC**				
NBH	TCS-based CS vs. phone-based CS	0.808	[0.657, 0.993]	0.043
	TCS-based CS vs. non-CS	0.544	[0.432, 0.683]	<0.001
	Phone-based CS vs. non-CS	0.673	[0.539, 0.84]	<0.001
Outside NBH	Phone-based CS vs. non-CS	0.608	[0.52, 0.709]	<0.001
**Door-to-needle for IV-tPA**				
NBH	TCS-based CS vs. phone-based CS	0.877	[0.694, 1.109]	0.264
	TCS-based CS vs. non-CS	0.383	[0.229, 0.641]	<0.001
	Phone-based CS vs. non-CS	0.437	[0.263, 0.724]	0.002
Outside NBH	Phone-based CS vs. non-CS	1.524	[0.667, 3.478]	0.307
**Door-to-puncture for EVT**				
NBH	TCS-based CS vs. phone-based CS	0.823	[0.466, 1.453]	0.467
	TCS-based CS vs. non-CS	0.63	[0.282, 1.408]	0.232
	Phone-based CS vs. non-CS	0.766	[0.308, 1.9]	0.531
Outside NBH	Phone-based CS vs. non-CS	0.641	[0.432, 0.951]	0.029

*The analysis on variances was performed using the logarithms of the door-to-CT time, door-to-CBC time, door-to-needle time for IV-tPA, and door-to-puncture time for EVT*.

*CI, confidence interval; CT, computed tomography; CBC, complete blood count; IV-tPA, intravenous tissue plasminogen activator; EVT, endovascular therapy*.

**Table 3:** The proportion of IV-tPA and EVT in patients treated with TCS-based CS or phone-based CS.

**IV-tPA**	***n*/all patients (%)**
TCS-based CS	21/27 (78)
Phone-based CS	12/26 (46)
**EVT**	
TCS-based CS	9/27 (33)
Phone-based CS	4/26 (15)

**Table 4:** The effects of using TCS for IV-tPA and EVT.

**Outcome**	**Odds ratio**	**95% CI**	***p*-value**
IV-tPA	3.98	[1.08, 14.59]	0.037
EVT	7.85	[0.4, 153.48]	0.174

*Models were adjusted for large vessel occlusion, oral anticoagulants, and the initial NIHSS score*.

### Results of the Effects of CS Itself on AIS Care

The key task processing times are shown in Figure 5. As for the patients treated during NBH, compared with the non-CS group, TCS-based CS and phone-based CS led to a significant reduction in the D2C time (β = 0.33, *p* < 0.001; β = 0.36, *p* < 0.001), the door-to-CBC time (β = 0.54, *p* < 0.001; β = 0.67, *p* < 0.001), and the DTN time (β = 0.38, *p* < 0.001; β = 0.44, *p* = 0.002). As for the patients treated outside of NBH, compared with the non-CS group, phone-based CS led to a statistically significant reduction in the following times: D2C time (β = 0.52, *p* < 0.001), door-to-CBC time (β = 0.61, *p* < 0.001), and D2P time (β = 0.64, *p* = 0.029) (Table 3).

Regarding the questionnaire survey, there were 34/38 (89%) valid responses (from 16 emergency nurses, 14 radiology technicians, and 4 laboratory technicians). In the phone-based CS group, problems such as the following were noted: repeated calling due to busy or unreachable phone lines; failing to notify other team members; redundant calling of the same department by mistake; calling a wrong phone number; and forgetting a phone number. In the TSC-based CS group, 82% (28/34) of respondents felt the communication burden to be reduced, compared to the phone-based CS group.

## Discussion

In the present study, the use of TCS in the parallel CS workflow significantly reduced door-to-CBC times, increased the rate of patients with IV-tPA, and, at the same time, lessened the burden of information sharing among the team members.

Several applications for AIS care have been developed earlier. These applications are excellent for image and text information sharing, but they do not have a multitask progress management function (13–18). Unlike these applications, TCS has been developed to support the management of tasks in AIS care. As the crucial tasks and the real-time progression of all tasks are always visible on the TCS dashboard, all stroke team members can process their own tasks while being aware of the real-time progress of other departments involved in AIS care.

In line with previous reports (5–10), this study confirms that the most effective factor that leads to a reduction in certain processing times in AIS care is the use of a CS algorithm itself. However, we show that the use of TCS in combination with a CS system has an additional time-saving effect. Furthermore, the result of the questionnaire survey showed that the use of TCS reduced the burden of information sharing within the AIS care team. We therefore think that when CS is introduced for the first time at another hospital, the addition of TCS would be an effective way of reducing treatment times and minimizing the communication burden among the team members.

The rate of IV-tPA was higher in the TCS-based CS group than in the phone-based CS group. Likewise, the rate of EVT was almost 2-fold in the TCS group compared to the non-TCS group. The exact reason for this is unclear, but we assume that, together with the reduction in the door-to-CBC time, the cohesion of the CS team, as an effect of the TCS, influenced the choice of the more aggressive treatment protocols.

In this study, we installed TCS in the ER, CT/MRI/angiography sites, laboratory, stroke care unit, and conference room and used it statically due to limited connection to the internet, thus requiring the personnel to remain at a specific location. Hence, improving internet access and availability could possibly further enhance the use of TCS 24/7.

Real-time feedback of time metrics to the stroke team members is essential to improve the quality of acute stroke care (19). However, the feedback process is time-consuming, and many institutions avoid it when possible (20). The performance history analyzer function of TCS can free the medical staff from the complicated tasks of recording times, calculating timelines, and writing performance reports. This function facilitates the identification of problems and the development of solutions in acute AIS care.

Our study has several limitations. First, the study design is retrospective in nature. It does not represent a continuous case series and has a relatively small sample size. Furthermore, selection bias and random errors may have affected the results. Second, the study was conducted at a single center. Third, that TCS was only used during NBH is a significant limitation of the study, because off-regular working hours and weekends are particularly limited in resources and personnel, and TCS would be of particular benefit during that period. Clinical trials that enable 24/7 use of TSC are thus required. Fourth, the statistical model for DTN times for IV-tPA showed slight overfitting. Therefore, the present results should be regarded as preliminary. Further prospective multicenter studies or clinical trials to confirm the 24/7 usefulness of TCS are recommended.

In conclusion, we developed a visual task management ICT application that supports parallel CS workflow in AIS care. In the single-center study described here, we show that using TCS shortens the processing times of some critical tasks and reduces the burden of information sharing among team members. TCS is a novel approach that enables rapid and stable stroke care. We therefore recommend our system to be applied in the care of patients with acute stroke worldwide.

## Data Availability Statement

All data sets generated for this study are included in the manuscript files.

## Ethics Statement

The studies involving human participants were reviewed and approved by The Ethics Committee of Kokura Memorial Hospital approved this study. Written informed consent for participation was not required for this study in accordance with the national legislation and the institutional requirements.

## Author Contributions

SM, HK, INak, and JKi conceived the design of the paper. INak, AI, THat, TO, MA, HC, WT, KTo, THashik, YF, TK, EH, SW, DK, AT, KF, THashim, JKo, KS, TT, HN, TI, RY, and INag contributed to the acquisition and analysis of the data. SM, HK, JKi, TO, KTa, and TI contributed to drafting the text and preparing the figures. All authors contributed to and approved the final manuscript.

### Conflict of Interest

The authors declare that the research was conducted in the absence of any commercial or financial relationships that could be construed as a potential conflict of interest.
